# Bow hunter’s syndrome due to an anomalous right vertebral artery origin and contralateral absence: a case report and literature review

**DOI:** 10.1186/s12883-024-03754-5

**Published:** 2024-07-12

**Authors:** Li Zhang, Yu Gao, Xiao Yu, Ying Guo, Zhe Piao, Guangxian Nan

**Affiliations:** 1 Department of Neurology, China-Japan Union Hospital, Jilin University, 126 Xiantai St., Changchun, Jilin 130033 China; 2 Department of Nephrology, China-Japan Union Hospital, Jilin University, 126 Xiantai St., Changchun, Jilin 130033 China

**Keywords:** Bow hunter’s syndrome, Rotational vertebral artery occlusion, Vertebral artery anomalies, Absence of left vertebral artery, Case report

## Abstract

**Background:**

Bow Hunter’s syndrome (BHS), also known as rotational vertebral artery occlusion (RVAO), is a rare condition characterized by dynamic vertebrobasilar insufficiency due to position-dependent occlusion of the vertebral artery (VA). In the existing literature, most cases of BHS are attributed to osteophytic compression originating from the occipital condyle or within the transverse foramen, often accompanied by anatomical abnormalities of the VA. However, cases presenting solely with VA anomalies in the absence of any cervical vertebral structural abnormality are rare. This case report presents a unique instance of BHS in a 56-year-old male, attributed to the anomalous origin of the right VA and the absence of the left VA, without cervical structural abnormalities.

**Case presentation:**

The patient exhibited symptoms like episodic dizziness and vertigo, which were exacerbated by rightward head rotation and alleviated upon returning to a neutral position. Diagnostic evaluation, including digital subtraction angiography, revealed that the right VA originated from the right common carotid artery and compression-induced stenosis of the right VA during head rotation. Conservative management, including avoidance of certain head movements and anti-arteriosclerosis medication, led to symptom resolution over a two-year follow-up period.

**Conclusions:**

This report contributes to the understanding of BHS by highlighting a rare vascular anomaly presentation and incorporates a review of 14 similar case reports in the literature describing that an anatomical abnormality of the VA is mainly responsible for the pathology of BHS in the absence of cervical vertebral anomalies, thus emphasizing the need for careful diagnostic and management strategies.

## Background

Bow Hunter’s Syndrome (BHS), also known as rotational vertebral artery occlusion (RVAO), is a rare condition marked by dynamic vertebrobasilar insufficiency (VBI) due to position-dependent vertebral artery (VA) occlusion. The clinical manifestations range from episodic dizziness to vertigo, potentially coupled with nystagmus, tinnitus, syncope, blurred vision, nausea, or vomiting [1]. These symptoms are typically alleviated by rotating the head to the neutral position. Diagnosis is most commonly established by dynamic digital subtraction cerebral angiography, wherein the patient is directed to rotate their head to provoke a similar symptomatic response, thereby determining the appropriate neck angle for angiographic analysis. Contrarily, static imaging techniques are generally ineffective for detecting position-related stenosis or occlusion [2].

In the existing literature, most cases of BHS are attributed to osteophytic compression originating from the occipital condyle or within the transverse foramen, often accompanied by anatomical abnormalities of the VA [3]. However, cases presenting solely with VA anomalies in the absence of any cervical vertebral structural abnormality are rare. The case presented herein represents a unique instance of RVAO characterized by the anomalous origin of the VA without cervical vertebral anomalies, compounded by the absence of the VA on the opposite side.

## Case presentation

A 56-year-old male presented with a one-year history of episodic dizziness and vertigo, often accompanied by nausea and vomiting. Symptoms typically manifested when turning his head to the right and subsided upon returning to a neutral position. Severe episodes included transient loss of consciousness. His medical history revealed a three-year history of gout. Physical examination showed a blood pressure discrepancy: 110/80 mmHg in the left arm and 140/90 mmHg in the right arm. The patient reported head fullness and dizziness with rightward head turns, which resolved when the head was in a neutral position. Vestibular function tests excluded otolithiasis and demonstrated transient vertical nystagmus.

**Fig. 1:**
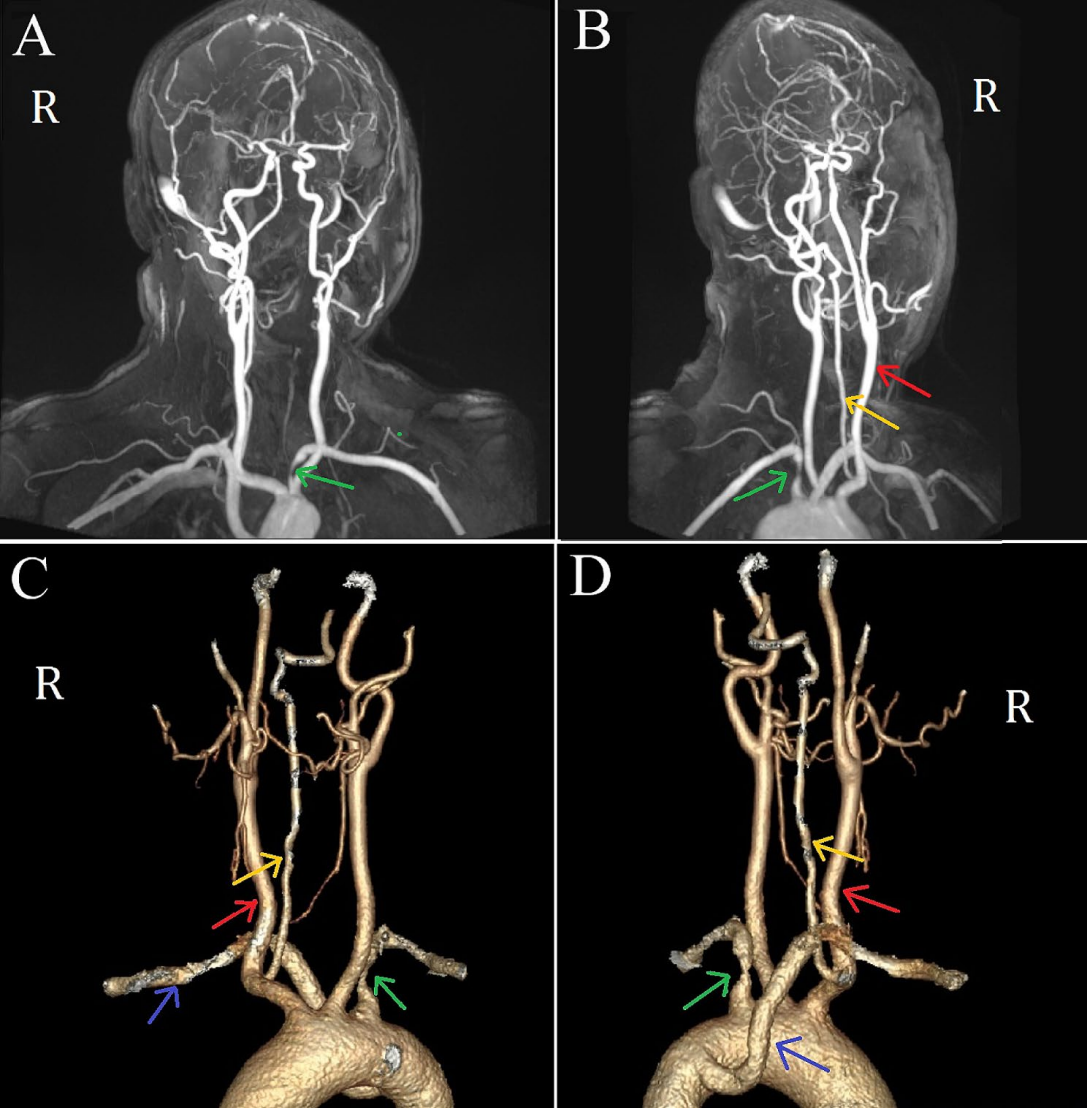
Magnetic resonance angiography (MRA) and computed tomography angiography (CTA) of the head and neck. Imaging reveals narrowing at the origin of the left subclavian artery and the absence of the left vertebral artery (VA). It also shows an aberrant right subclavian artery and the right VA originating from the right common carotid artery (**A** Anterior view of MRA, **B** Lateral view of MRA, **C** Anterior view of CTA, **D** Lateral view of CTA). Red arrows indicate the right internal jugular vein, yellow arrows indicate the right VA, green arrows indicate the left subclavian artery, and blue arrows indicate the aberrant right subclavian artery

**Fig. 2:**
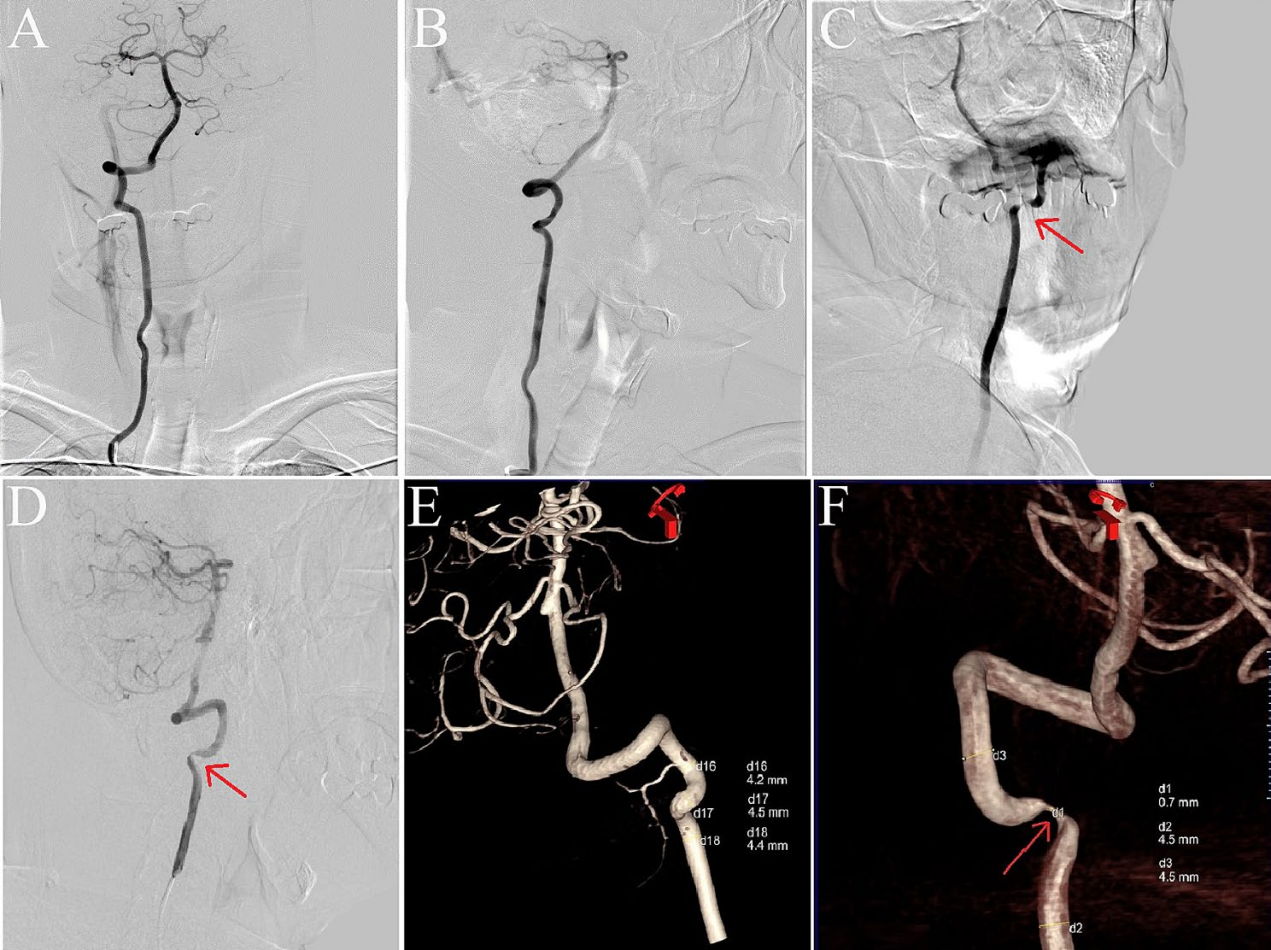
Digital subtraction angiography imaging. With the head in a neutral position, angiography of the vertebral artery (VA) shows no stenosis (**A** Anterior view; **B** Lateral view). When the head is turned to the right, angiography of the right VA reveals stenosis, during which the patient experiences dizziness and returns to a neutral position (**C** Anterior view; **D** Lateral view). Three-dimensional angiographic imaging of the right VA is shown in the neutral (**E**) and right-turned head positions (**F**). The red arrows indicate the site of vascular stenosis

**Fig. 3:**
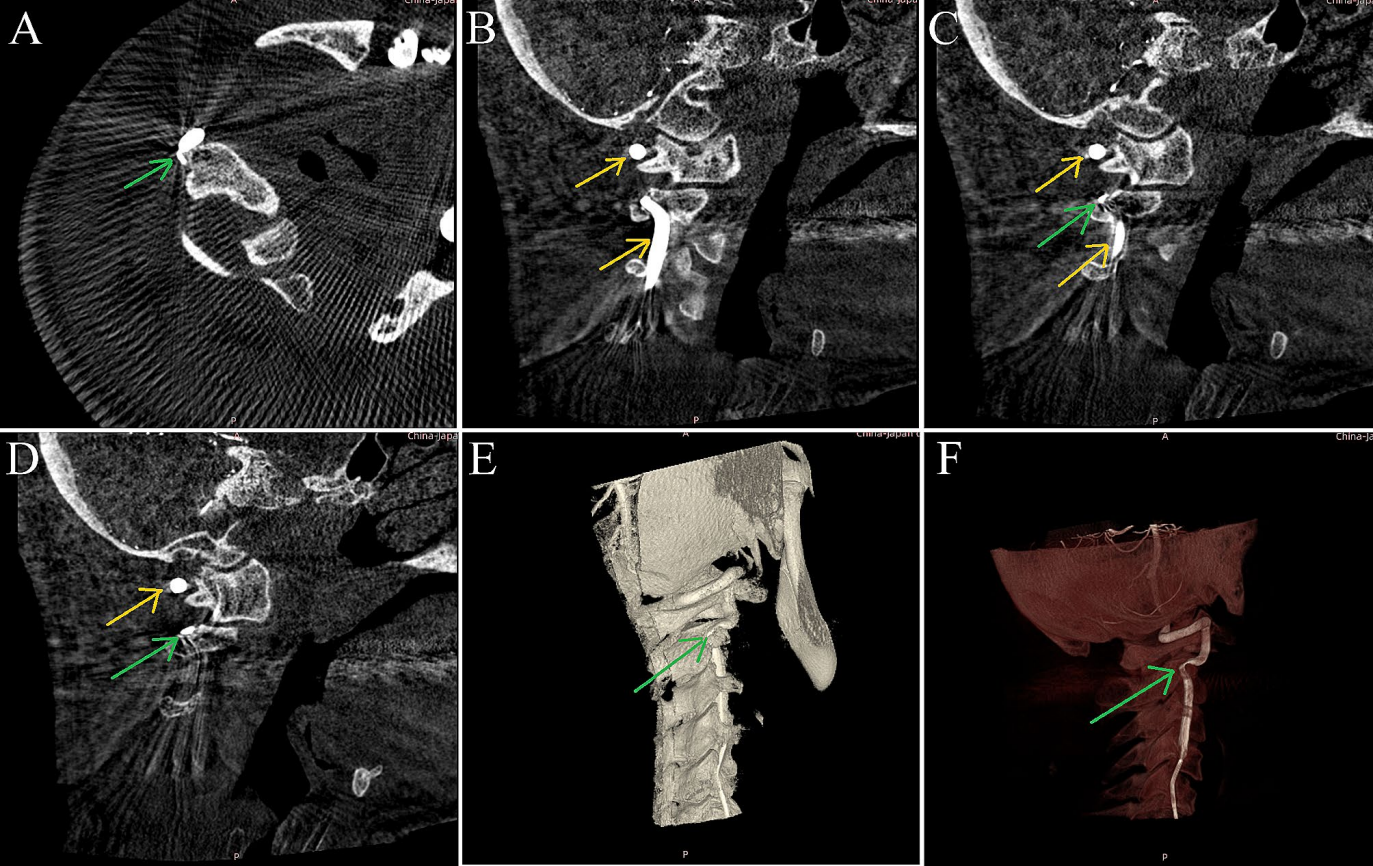
The right vertebral artery (VA) is susceptible to compression by the C2 transverse processes during head and neck rotation. **A**, **B**, **C**, **D** Cross-sectional digital subtraction angiography (DSA) images, with yellow arrows indicating the normal VA lumen and green arrows pointing to the narrowed segment of the vessel. **E**, **F** Three-dimensional DSA-magnetic resonance fusion image, where green arrows indicate the narrowed segment of the VA lumen

Head computed tomography and magnetic resonance imaging (MRI) scans conducted upon admission revealed no significant abnormalities. Neck vascular ultrasound indicated a reduced blood flow velocity in the right VA with changes in the head position. Head and neck magnetic resonance angiography and neck computed tomography angiography identified the features of intracranial posterior circulation, showing that the right VA originated from the right common carotid artery, while the left VA was absent. The right VA directly extended into the basilar artery, which then branched into normal bilateral posterior cerebral arteries, with no fetal-type posterior cerebral artery observed. There was no abnormality detected in the circle of Willis (Fig. 1). Cervical spine X-rays and MRI showed no evidence of a bony abnormality or tumor. Digital subtraction angiography in a neutral position revealed that the right VA entered the transverse foramen at C6 and had no stenosis (Fig. 2). However, when the patient turned his head to the right, stenosis in the right VA was observed as it exited the C2 foramen, coinciding with episodes of dizziness (Fig. 2). Further analysis indicated that a rightward head rotation led to compression of the right VA by the C2 transverse process (Fig. 3).

**Fig. 4:**
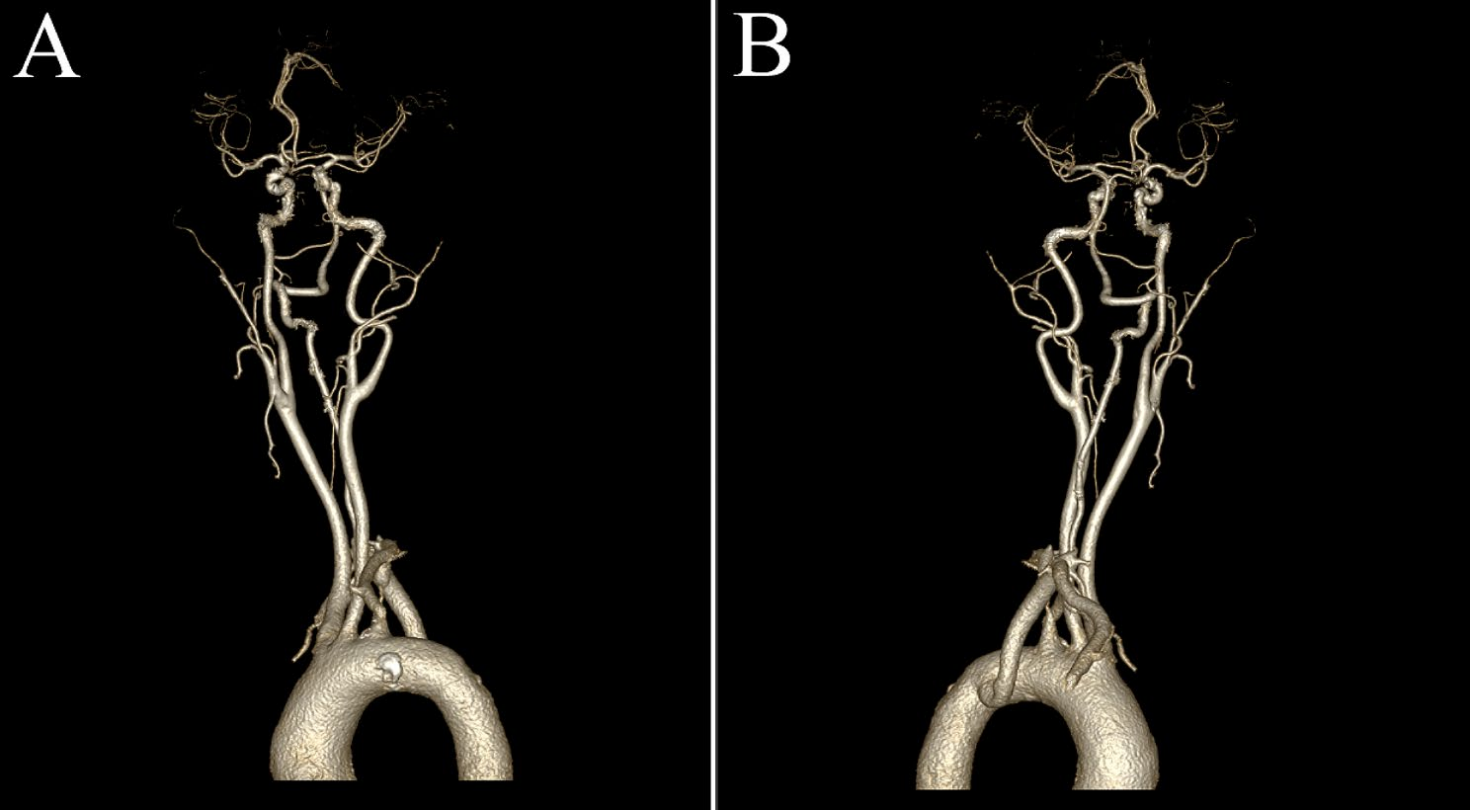
Follow-up computed tomography angiography reveals no stenosis at the compression site of the right vertebral artery (**A** Anterior view; **B** Lateral view)

Based on the clinical symptoms and radiological evidence, BHS was diagnosed and attributed to the anomalous origin of the dominant right VA and the absence of the left VA. The patient underwent a multidisciplinary consultation, including experts in neurology, spinal surgery, and interventional neuroradiology. The spinal surgery expert suggested upper cervical spine fusion surgery, while the interventional neuroradiology expert proposed considering laser-carved nitinol stent implantation. However, both experts indicated that surgical treatment should only be considered if medical therapy proves ineffective, and it is not the first-line treatment option. Finally, the patient chose a conservative management approach. He was advised to minimize excessive rightward neck rotation. Considering his coexisting arteriosclerosis, he was prescribed oral atorvastatin, aspirin, and probucol for anti-arteriosclerotic therapy, accompanied by regular monitoring of liver function and routine blood biochemistry. After a two-year follow-up period, the patient reported no further episodes of vertebrobasilar insufficiency. Subsequent head and neck computed tomography angiography revealed no progression of vascular stenosis (Fig. 4).

## Discussion and conclusions

BHS is not age-specific, though it predominantly occurs in individuals aged between 50 and 70 years old, with males being more frequently affected than females [4]. The precise incidence of BHS remains unclear due to the scarcity of case reports and series. To date, a total of 239 cases reported with RVAO or BHS have been reported. The infrequency of this condition has led to a lack of established protocols for its diagnosis and treatment. The primary approaches for managing this syndrome include conservative treatments, surgical intervention, and endovascular procedures.

BHS typically involves the dominant VA being mechanically compressed against bony components during head rotation at the atlantoaxial junction. Additionally, in the subaxial cervical region, similar compression may occur due to osteophytic growths or fibrous structures. VA anomalies and their tortuosity, cervical spondylosis and related structural irregularities, instability in the atlantoaxial area, and cervical muscle contraction are also considered as additional etiological factors contributing to the development of BHS [5]. It has been reported that vascular causes (aneurysm, VA hypoplasia, other vascular malformations, VA dissection, atherosclerosis, or embolism) account for 5% of etiologies [5]. Here, we have reviewed 14 case reports in the literature in which an anatomical abnormality of the VA is mainly responsible for the pathology of BHS in the absence of cervical vertebral anomalies (Table 1). The VA is known for having a variable anatomy, which primarily includes anomalies of origin or course, abnormal diameter (hypoplasia), or fenestration.

**Table 1:** Reported cases of Bow Hunter’s syndrome with anatomical abnormalities of the VA or the absence of cervical vertebral anomalies

Study	Age, sex	Anatomical abnormality of VA	Mechanism of compression	Symptoms	Therapy	Outcome and follow-up
Johnson et al. [7]	42, M	Anomalous course of the right VA, which entered the transverse foramen at C4.	Preforaminal compression by the anterior tubercle of the C5 transverse process.	Loss of color vision and disorientation while rotating his neck to the right.	Decompression of the VA by resecting the anterior C5 tubercle.	After an eight-month follow-up, he has had no further ischemic events and has no restrictions with physiologic neck movement.
Wakayama et al. [9]	45, F	Kinking and stretching of the left VA at the C2 foramina dissection of the right VA.	Constriction of the left VA was speculated to be caused by compression of both the transverse process of C2 and the intertransversarius muscle.	Pain around the posterior part of the neck on the right side, and then dizziness when turning the head to the right side.	Conservatively with voluntary restriction of her head movement, diet, and antihypertensive therapy with amlodipine besilate and celiprolol hydrochloride.	The six-month follow-up showed neither exacerbation nor recurrence.
Motiei-Langroudi et al. [10]	61, M	A tortuosity in the left VA, and complete occlusion in the V1 segment when the head was rotated to the left. Right VA hypoplasia terminating in the PICA.	With the patient’s head turned to the left, the tortuous loops of the left VA were pushed together even closer, resulting in occlusion of the VA.	Lightheadedness and facial numbness triggered by turning his head to the left.	Self-expanding biliary stent placement.	After follow-up for 3.5 months, the patient was free of symptoms, and there was no dynamic occlusion in the proximal VA in angiography.
Rendon et al. [11]	76, M	Completely extraosseous V2 segment of the right VA, and hypoplastic left VA.	Narrowing of the right VA caused by extrinsic compression from thyroid cartilage when the head was rotated to the right.	Near-syncope on rightward head rotation resulting in two falls.	Surgical correction with arteriolysis of the VA and resection of the adjacent compressive structures.	Continues to be asymptomatic eight months after surgery.
Hong et al. [8]	29, M	Right VA with an aberrant course entered the foramen transversarium at C4. The left VA was hypoplastic.	Complete right VA occlusion at C6 was felt to be secondary to compression between the longus colli and the longus capitis muscles against an enlarged anterior C6 tubercle of the transverse process.	Recurrent vertigo and syncope with right rotation and extension of the neck.	Surgical decompression of the right VA at C5–C6 and laryngoplasty.	The patient has not had any further syncopal episodes at more than two years post-surgery.
Tanaka et al. [22]	56, M	Aberrant course of the left VA, mild narrowing, and endothelial irregularity through the V3 segment.	Hyperdynamic, repetitive head turning movements likely caused endothelial disruption and narrowing of the V3 segment of the artery with slowed blood flow.	Two episodes of visual blurriness, dizziness, and nausea while swinging golf clubs.	Endovascular sacrifice of the VA.	No further symptom recurrence with any degree of head turning at the six-month follow-up.
Kitahara et al. [14]	83, M	Hypoplastic left VA, severe stenosis of the right VA with head rotation.	The hypoplastic left VA was occluded by a thrombus extending from the left subclavian artery ligation site, and dynamic stenosis of the right VA by head rotation induced dizziness.	Dizziness when turning his head to the left.	Managed conservatively.	Not mentioned.
Kan et al. [15]	41, M	The right VA ended in the PICA, and the left VA was of good caliber. Chronic dissection and stasis of flow in the right VA when the head was rotated to the right.	Head rotation then led to flow impedance in the ipsilateral VA, likely predisposed by his previous traumatic dissection, causing hypoperfusion of the posterior circulation resulting in symptoms of severe VBI.	Severe VBI, including bradycardia, nausea, vomiting, and vertigo when he rotated his head to the right.	PICA-to-PICA in situ bypass.	complete resolution of his vertebrobasilar symptoms
Nomura et al. [16]	47, F	Right VA hypoplasia.	The blood flow of the right VA was less than that of the left VA when the neck was rotated leftward, and further reduction of blood flow was seen. In addition, the systolic blood pressure was too low.	Vertigo, nausea, and dullness of the right arm when she turned her face to the left.	Conservative therapy and avoid rotating the neck leftward in daily life.	Her vertigo was cured by the modification of a prescription for her past medical history: hypertension.
Yamaguchi et al. [21]	45, M	Stretching and sliding of the left VA fenestration, and the lower limb was compressed between the C1 transverse process and the C2 lateral mass when the head was rotated to the right.	Excessive rotation stretched the VA, and this VA was compressed between the C1 posterior arch and the C2 lateral mass because of space narrowing caused by excessive rotation.	Repeated vertigo associated with head rotation to the right for two years.	C1/C2 posterior fusion using C1/C2 transarticular screws and a bone graft.	No symptoms or recurrent strokes were observed at 24 months after the surgery.
Noh et al. [23]	59, M	Hypoplastic right VA terminating in the PICA, and complete occlusion when the head was rotated to the left.	Not mentioned.	Vertigo and nystagmus induced by leftward head rotation.	Not mentioned.	Not mentioned.
Hernandez et al. [24]	49, F	A pseudoaneurysm at the left VA V3 segment and near total occlusion as it exited the C2 foramen.	There was an associated pseudoaneurysm of the V3 segment distal to the site of a rotational mechanical obstruction.	Vague neck pain and severe vertigo, nausea, and near syncope when her head turned up and to the right.	Computed tomography-navigated posterior instrumentation using bilateral C1 lateral mass screws, C2 translaminar screws, and bilateral intrafacet cages.	A six-month follow-up, MRA demonstrated a stable size and shape of the pseudoaneurysm, and she was doing well without complaints.
Sadeghipour et al. [25]	24, F	A large aberrant VA originated from the proximal landing zone of the aortic arch.	Suspicious compression by an aortic pseudoaneurysm.	Episodes of syncope and dizziness.	Endovascular solutions.	Nonrecurrence of syncopal attacks after about nine months.
Strupp et al. [26]	48, M	Complete occlusion of the right VA at C2 with head rotation to the left. 60–70% stenosis at the left VA origin.	Not mentioned.	Severe rotatory vertigo and tinnitus in the right ear, accompanied by torsional downbeat nystagmus.	Right VA was decompressed from C2 up to its penetration through the dura.	The patient was free of symptoms, even during extreme rotations of the head toward the left.
Present case	50, M	The left VA is absent, and the right VA originates from the right common carotid artery.	The right VA is susceptible to compression by the C2 transverse processes during head and neck rotation.	Dizziness while rotating his neck to the right.	Avoid excessive turning of the neck to the right. Take atorvastatin and aspirin probucol tablets orally.	The patient was followed up for two years without obvious symptoms.

MRA, magnetic resonance angiography; PICA, posterior inferior cerebellar artery; VA, vertebral artery; VBI, vertebrobasilar insufficiency

### Origin or course anomalies of the VA

A recent systematic review assessed the prevalence of various VA variations [6]. The predominant origin for both VAs was found to be the subclavian artery. The left VA originating from the aortic arch was reported to occur in 4.81% of cases. Additional origins for the right VA include the aortic arch (0.1%), right common carotid artery (0.1%), and brachiocephalic trunk (0.5%).

Previous cases illustrate a variety of aberrations in origin or course of the VA, contributing significantly to the pathologies of BHS. For instance, Johnson et al. [7] reported a case in which the anomalous course of the right VA entering the transverse foramen at C4 led to preforaminal compression by the anterior tubercle of the C5 transverse process, inducing ischemic symptoms upon neck rotation to the right. Similarly, Hong et al. [8] described a case in which the aberrant entry of the right VA at C4 resulted in complete occlusion at C6 due to compression between the neck muscles against an enlarged anterior tubercle. Furthermore, Wakayama et al. [9] observed kinking and stretching of the VA at the C2 foramina, with speculated compression by the transverse process of C2 and the intertransversarius muscle, leading to neck pain and dizziness. In addition, Motiei-Langroudi et al. [10] identified a case with a tortuous left VA, with complete occlusion in the V1 segment upon head rotation to the left, emphasizing the dynamic nature of these compression events. Moreover, Rendon et al. [11] presented a unique instance of extrinsic compression of the right VA by thyroid cartilage, causing near-syncope on rightward head rotation. Cervical vertebral anomalies were not found in any of the above cases. These cases highlight how atypical VA trajectories can directly contribute to the onset of BHS, leading to dynamic occlusions.

During head rotation, the VA intertransverse segment is stretched due to the increased distance between the transverse foramina, while its intracranial segment remains fixed. Additionally, the sharp trajectory of the VA after exiting the C2 foramen makes it vulnerable to strain and compression. In this patient, the right VA originated from the common carotid artery and had a shorter course; therefore, it was more susceptible to continuous tension or compression by surrounding tissues, such as muscles, during head rotation, leading to its compression at the C2 foramen exit. The anomaly in the origin of the right VA may be a congenital developmental abnormality. However, considering that this patient did not present with posterior circulation symptoms until the age of 56 years, we propose the following explanation: with aging, the elastic fibers in the arterial wall are progressively replaced by collagen fibers, resulting in decreased arterial elasticity. This reduction in elasticity makes the vertebral artery more susceptible to compression and stenosis during movements such as head rotation. The symptoms of this patient were relieved by anti-atherosclerosis medications, including atorvastatin, aspirin, and probucol. Collectively, these medications contribute to the amelioration of arterial stiffness and enhance vascular compliance.

### Hypoplasia of the VA

The normal diameter of the VA ranges from 3 mm to 5 mm, and it is not always symmetrical on both sides. Currently, no uniform standard exists for diagnosing VA hypoplasia, but common criteria include the following: cervical vascular ultrasound showing a blood flow of less than 40 mL/min, a diameter of less than 2.5 mm, or bilateral asymmetry exceeding 1:1.7 [12]; or cervical MRI revealing a diameter of less than 2 mm [13]. VA hypoplasia is reported in 7.94% of vessels [6].

Recent case studies highlight the crucial role of VA hypoplasia in the development of BHS. Kitahara et al. [14] documented an 83-year-old male with a hypoplastic left VA and severe right VA stenosis with head rotation to the left. The hypoplastic left VA was occluded by a thrombus from the left subclavian artery ligation site, and dynamic stenosis of the right VA during head rotation caused dizziness, which was managed conservatively. In addition, Kan et al. [15] reported a 41-year-old male whose right VA ended in the posterior inferior cerebellar artery (PICA), with chronic dissection and flow stasis during right head rotation, managed successfully with a PICA-to-PICA in situ bypass. Nomura et al. [16] described a 47-year-old female with right VA hypoplasia. When her neck was rotated leftward, the blood flow in her right VA decreased significantly, coupled with a low systolic blood pressure. Conservative therapy and lifestyle adjustments effectively cured her vertigo. In fact, most patients with BHS have a hypoplastic contralateral VA along with dominant VA occlusion, resulting in symptoms of VBI on head rotation [8, 10, 11].

Moreover, the congenital absence of the VA is classified as a rare form of hypoplasia. Few cases with the absence of a unilateral VA or bilateral VAs have been reported, most of which presented with symptoms [17–19]. In this case, the absence of the contralateral VA means that when the dominant artery is narrowed, the other side cannot compensate effectively, resulting in an insufficient blood supply from the VA and symptoms of posterior cerebral circulation ischemia.

### VA fenestration

An extracranial VA fenestration, a notable anatomical variation, is most often identified at the C1/C2 level. This fenestration typically consists of an upper limb, extending between the occipital bone and C1, and a lower limb, spanning from C1 to C2, detected in approximately 0.5–0.9% of patients [20]. Only one case of BHS due to VA fenestration has been reported [21]. This case involved a 45-year-old man with BSH due to congenital VA fenestration stretching and sliding between C1 and C2 after rightward head rotation. Excessive rotation caused the VA to be stretched and compressed due to the narrowing space between the C1 posterior arch and the C2 lateral mass. Treatment involved C1/C2 posterior fusion using C1/C2 transarticular screws and a bone graft, which proved to be effective.

Collectively, these cases highlight the varied and significant impact of VA anomalies in the mechanism of BHS, necessitating careful consideration in both diagnostic and therapeutic interventions. They underscore the importance of individualized management strategies, ranging from conservative treatment to surgical interventions, depending on the severity and specifics of the vascular anomalies. To the best of our knowledge, this is the first report of BHS due to the anomalous origin of the dominant right VA along with the absence of the contralateral VA. The repetitive compression of the vessel in this patient significantly heightened the risk of endothelial injury and thrombosis, emphasizing the potential for stroke in similar cases. The successful management through conservative treatment and careful monitoring underlines the importance of recognizing and addressing these rare presentations of BHS. This case serves as a critical reminder of the diverse etiologies and presentations of BHS, necessitating thorough evaluation and individualized management strategies for each patient.

## Data Availability

The datasets used and/or analysed during the current study are available from the corresponding author on reasonable request.

## References

[1] Luzzi S, Gragnaniello C, Marasco S, Lucifero AG, Del Maestro M, Bellantoni G (2021). Subaxial vertebral artery rotational occlusion syndrome: an overview of clinical aspects, Diagnostic Work-Up, and Surgical Management. Asian Spine J.

[2] Rastogi V, Rawls A, Moore O, Victorica B, Khan S, Saravanapavan P (2015). Rare etiology of Bow Hunter’s syndrome and systematic review of literature. J Vasc Interv Neurol.

[3] Duan G, Xu J, Shi J, Cao Y (2016). Advances in the Pathogenesis, diagnosis and treatment of Bow Hunter’s syndrome: a Comprehensive Review of the literature. Interv Neurol.

[4] Jost GF, Dailey AT (2015). Bow hunter’s syndrome revisited: 2 new cases and literature review of 124 cases. Neurosurg Focus.

[5] Schulz R, Donoso R, Weissman K (2021). Rotational vertebral artery occlusion (bow hunter syndrome). Eur Spine J.

[6] Tudose RC, Rusu MC, Hostiuc S. The vertebral artery: a systematic review and a Meta-analysis of the current literature. Diagnostics (Basel). 2023;13. 10.3390/diagnostics13122036 PMC10296927 37370931

[7] Johnson SA, Ducruet AF, Bellotte JB, Romero CE, Friedlander RM (2017). Rotational vertebral artery dissection secondary to anomalous entrance into transverse foramen. World Neurosurg.

[8] Hong X, D’Heygere E, Prisman E (2023). Thyroid cartilage Compression Causing Bow Hunter’s syndrome. Ann Otol Rhinol Laryngol.

[9] Wakayama K, Murakami M, Suzuki M, Ono S, Shimizu N (2005). Ischemic symptoms induced by occlusion of the unilateral vertebral artery with head rotation together with contralateral vertebral artery dissection–case report. J Neurol Sci.

[10] Motiei-Langroudi R, Griessenauer CJ, Alturki A, Adeeb N, Thomas AJ, Ogilvy CS (2017). Bow Hunter’s syndrome from a tortuous V1 segment vertebral artery treated with Stent Placement. World Neurosurg.

[11] Rendon R, Mannoia K, Reiman S, Hitchman L, Shutze W (2019). Rotational vertebral artery occlusion secondary to completely extraosseous vertebral artery. J Vasc Surg Cases Innov Tech.

[12] Chen YY, Chao AC, Hsu HY, Chung CP, Hu HH (2010). Vertebral artery hypoplasia is associated with a decrease in net vertebral flow volume. Ultrasound Med Biol.

[13] Park JH, Kim JM, Roh JK (2007). Hypoplastic vertebral artery: frequency and associations with ischaemic stroke territory. J Neurol Neurosurg Psychiatry.

[14] Kitahara H, Takeda T, Akasaka K, Kamiya H (2017). Bow Hunter syndrome elicited by vertebral arterial occlusion after total arch replacement. Interact Cardiovasc Thorac Surg.

[15] Kan P, Yashar P, Langer DJ, Siddiqui AH, Levy EI (2012). Posterior inferior cerebellar artery to posterior inferior cerebellar artery in situ bypass for the treatment of Bow hunter’s-type dynamic ischemia in holovertebral dissection. World Neurosurg.

[16] Nomura Y, Toi T. Transitional nystagmus in a Bow Hunter’s Syndrome case report. 2020;20:435. 10.1186/s12883-020-02009-3 PMC7706255 33256636

[17] Montechiari M, Iadanza A, Falini A, Politi LS (2013). Monolateral type I proatlantal artery with bilateral absence of vertebral arteries: description of a case and review of the literature. Surg Radiol Anat.

[18] Tsukamoto S, Hori Y, Utsumi S, Tanigake T, Horiike N, Otani R (1981). Proatlantal intersegmental artery with absence of bilateral vertebral arteries. Case report. J Neurosurg.

[19] Franks AJ, Hiss J, Sivaloganathan S (1987). Congenital absence of the left vertebral artery with traumatic thrombosis of the right artery. Med Sci Law.

[20] Hong JT, Lee SW, Son BC, Sung JH, Yang SH, Kim IS (2008). Analysis of anatomical variations of bone and vascular structures around the posterior atlantal arch using three-dimensional computed tomography angiography. J Neurosurg Spine.

[21] Yamaguchi S, Horie N, Tsunoda K, Tateishi Y, Izumo T, Hayashi K (2015). Bow Hunter’s stroke due to stretching of the vertebral artery fenestration: a Case Report. NMC Case Rep J.

[22] Tanaka K, Steinfort B. Rare cause of Bow Hunter’s syndrome due to an aberrant course of a vertebral artery. BMJ Case Rep. 2019;12. 10.1136/bcr-2019-229584 PMC6663298 31340945

[23] Noh Y, Kwon OK, Kim HJ, Kim JS (2011). Rotational vertebral artery syndrome due to compression of nondominant vertebral artery terminating in posterior inferior cerebellar artery. J Neurol.

[24] Hernandez RN, Wipplinger C, Navarro-Ramirez R, Patsalides A, Tsiouris AJ, Stieg PE (2019). Bow Hunter Syndrome with Associated Pseudoaneurysm. World Neurosurg.

[25] Sadeghipour P, Shafe O, Pouraliakbar H, Moosavi J (2018). Aberrant vertebral artery: an intruder into the aortic Arch (atypical Bow Hunter’s syndrome). JACC Cardiovasc Interv.

[26] Strupp M, Planck JH, Arbusow V, Steiger HJ, Bruckmann H, Brandt T (2000). Rotational vertebral artery occlusion syndrome with vertigo due to labyrinthine excitation. Neurology.

